# Management of Complications After Pancreaticoduodenectomy: A Narrative Review of Pathophysiology and Treatment Strategies

**DOI:** 10.7759/cureus.101919

**Published:** 2026-01-20

**Authors:** Gevorg Manoukian, Muzammil R Qureshi, Arsalan Bangash, Yaseen Hussain, Jyoti Singh, Gonzalo Navarro, Archana Acharya, Aliza Abid, Tazeen Fatima, Ammara Saleem, Minahil Saleem, Manju Rai

**Affiliations:** 1 Medicine, St. George’s University School of Medicine, St. George's, GRD; 2 Clinical Sciences, Caribbean Medical University School of Medicine, Willemstad, CUW; 3 General Medicine, Northampton General Hospital, Northampton, GBR; 4 Acute Medicine, Northampton General Hospital, Northampton, GBR; 5 Medicine, American University of Barbados, Bridgetown, BRB; 6 Internal Medicine, Servicio de Cirugia General del Hospital Universitario Fundacion Favaloro, Ciudad Autónoma de Buenos Aires (CABA), ARG; 7 General Surgery, Sri Ramachandra Bhanja Medical College, Cuttack, IND; 8 Internal Medicine, University of Medicine and Health Sciences, Basseterre, KNA; 9 Internal Medicine, Jinnah Medical and Dental College, Karachi, PAK; 10 Internal Medicine, Rashid Latif Medical College, Lahore, PAK; 11 Biotechnology, Shri Venkateshwara University, Gajraula, IND

**Keywords:** bile duct leaks, delayed gastric emptying, interventional radiology, intraabdominal abscess, pancreatic fistula, pancreatic insufficiency, pancreaticoduodenectomy, postoperative complications, postoperative hemorrhage, surgical wound infection

## Abstract

Pancreaticoduodenectomy (PD), or the Whipple procedure, remains the cornerstone operation for malignant and select benign diseases of the pancreatic head and periampullary region. Despite advances in surgical techniques and perioperative management, PD continues to be associated with high morbidity. Common complications such as postoperative pancreatic fistula (POPF), delayed gastric emptying (DGE), and postpancreatectomy hemorrhage (PPH) significantly influence recovery, healthcare costs, and long-term outcomes. A comprehensive literature review was conducted using PubMed/MEDLINE, Embase, Scopus, and Cochrane Library databases from inception to August 2025. Relevant studies, meta-analyses, clinical trials, and consensus guidelines in English were included if they addressed the incidence, pathophysiology, risk factors, diagnosis, management, or prevention of complications following PD. Data were synthesized to summarize contemporary management approaches and emerging innovations. POPF, DGE, and PPH remain the most frequent and clinically impactful complications. Advances in interventional radiology, including percutaneous drainage and endovascular embolization, have reduced reoperation rates and mortality. Supportive care-encompassing nutritional optimization, infection control, and prokinetic therapy-forms the foundation of management. Preventive strategies such as Enhanced Recovery After Surgery (ERAS) pathways, selective somatostatin analogue use, and early drain removal have shown improved outcomes. Emerging areas include artificial intelligence (AI)-based predictive models, omics-derived biomarkers, and minimally invasive or hybrid interventions.

Effective management of PD complications requires a multidisciplinary, evidence-based, and patient-centered approach. Integration of interventional, pharmacologic, and preventive strategies, combined with predictive technologies and standardized definitions, is essential to reduce morbidity and enhance survival after PD.

## Introduction and background

**Figure 1:**
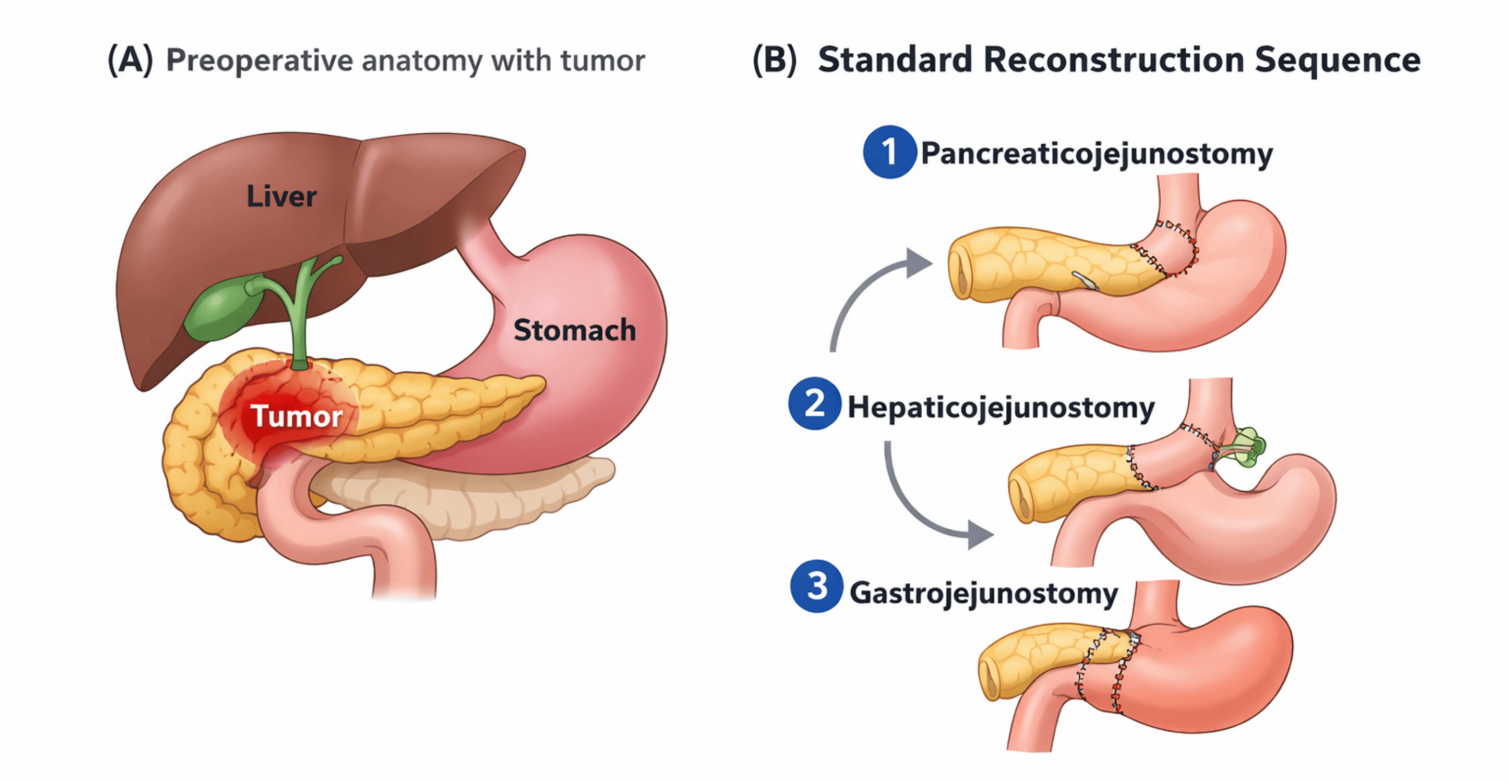
Schematic representation of the Whipple procedure: (A) preoperative anatomy with tumor in the pancreatic head and (B) postoperative reconstruction following the standard sequence-pancreaticojejunostomy (PJ), hepaticojejunostomy (HJ), and gastrojejunostomy (GJ). While variations in reconstruction order may be adopted in selected intraoperative scenarios, the figure depicts the commonly followed standard approach. This is an original image created by the author Gevorg Manoukian

Pancreaticoduodenectomy (PD), commonly referred to as the Whipple procedure, remains the mainstay surgical intervention for malignant and select benign diseases involving the pancreatic head, periampullary region, and distal bile duct [1]. Despite significant advances in surgical techniques, anesthesia, and perioperative care, PD continues to be regarded as one of the most technically demanding abdominal operations. The intricate anatomy of the pancreas, its proximity to major vascular structures, and the need for complex gastrointestinal reconstructions contribute to the high operative complexity and associated risk [2]. The anatomical complexity of PD is illustrated in Figure 1, depicting the standard sequence of reconstruction after PD, consisting of pancreaticojejunostomy (PJ) followed by hepaticojejunostomy (HJ) and gastrojejunostomy (GJ).

The incidence of postoperative complications following PD remains considerable, even in high-volume centers. Reported morbidity rates range between 30% and 60%, while mortality has significantly declined to below 5% in specialized centers [3,4]. Complications such as postoperative pancreatic fistula (POPF), delayed gastric emptying (DGE), postpancreatectomy hemorrhage (PPH), biliary leaks, and infectious complications continue to pose significant challenges [5]. Reported incidence rates vary, with POPF occurring in approximately 10%-30%, DGE in 15%-45%, and PPH in 3%-10% of patients [4]. These events not only prolong hospital stays and increase healthcare costs but also adversely impact long-term oncologic outcomes, patient quality of life, and survival [6,7].

Reconstruction following PD most commonly involves PJ or pancreaticogastrostomy (PG), combined with biliary and gastric reconstruction, with technique selection influenced by gland texture, duct size, and surgeon experience. To facilitate uniform reporting and comparison across studies, standardized definitions proposed by the International Study Group of Pancreatic Surgery (ISGPS) are widely adopted for complications such as POPF, DGE, and PPH [8,9]. POPF is defined as a drain output of any measurable volume with an amylase level greater than three times the upper limit of normal serum amylase, with clinically relevant grades B and C associated with increased morbidity. DGE is classified based on the duration of nasogastric decompression and the inability to tolerate oral intake, and is graded from A to C according to clinical severity. PPH is categorized by timing (early or late), location (intraluminal or extraluminal), and severity, guiding diagnostic and therapeutic decision-making. Awareness of these definitions and complication profiles is essential for interpreting outcomes and guiding evidence-based management strategies.

A thorough understanding of the underlying pathophysiology of each complication is critical for devising preventive and therapeutic strategies. For instance, the risk of POPF is influenced by gland texture, duct size, and anastomotic techniques, tumor pathology and location, use of neoadjuvant therapy, intraoperative blood loss, and overall operative complexity, whereas DGE is often multifactorial, involving both mechanical and functional etiologies [7,8]. Similarly, hemorrhagic complications may result from vascular erosion or pseudoaneurysm formation, highlighting the importance of early recognition and tailored surgical or interventional radiologic management [9]. Individualized management approaches, often involving a combination of surgical, endoscopic, radiologic, and pharmacologic measures, are essential for optimizing patient outcomes [10].

Accordingly, the primary aim of this narrative review is to provide a comprehensive overview of the pathophysiology, risk factors, and evidence-based management of complications following PD. The review is structured by major complication type, with focused discussion on POPF, DGE, PPH, and other clinically relevant sequelae, followed by sections outlining supportive care, interventional radiology (IR), surgical reintervention, preventive strategies, and emerging approaches. By integrating current knowledge, we seek to aid clinicians in delivering patient-centered, complication-specific care while identifying areas that warrant further research and innovation.

## Review

Methodology

The literature search for this narrative review was carried out using PubMed/MEDLINE, Embase, Scopus, and the Cochrane Library from January 2006 to August 2025. A combination of Medical Subject Headings (MeSH) and free-text keywords was employed to identify relevant studies. The primary MeSH terms and keywords included pancreaticoduodenectomy, Whipple procedure, pancreatic surgery, postoperative complications, pancreatic fistula, delayed gastric emptying, postpancreatectomy hemorrhage, bile leak, chyle leak, intra-abdominal abscess, surgical site infection, pancreatic insufficiency, interventional radiology, endovascular procedures, Enhanced Recovery After Surgery (ERAS), somatostatin analogues, proton pump inhibitors, artificial intelligence, predictive models, and biomarkers. Boolean operators “AND” and “OR” were applied to refine the search. Additionally, reference lists of relevant systematic reviews and key articles were screened to capture any studies missed in the initial search. A final reference audit was performed prior to submission to ensure that all cited studies were accurate, up to date, and directly relevant and appropriately supported the statements made in the review.

Inclusion criteria were peer-reviewed original studies, systematic reviews, meta-analyses, clinical trials, and consensus guidelines published in English that addressed the incidence, risk factors, pathophysiology, diagnosis, management, or prevention of complications following PD. Studies involving adult patients (≥18 years) undergoing PD for malignant or benign conditions were considered, provided they reported on surgical, endoscopic, radiologic, pharmacologic, or supportive management strategies. Pediatric populations (<18 years) were excluded due to differences in disease spectrum, surgical indications, and perioperative outcomes. Additional exclusion criteria included case reports or case series with fewer than 10 patients, non-English publications without available translations, studies focused solely on distal or total pancreatectomy unless comparative with PD, and animal or cadaveric studies. Titles and abstracts were screened independently by two reviewers, with full texts assessed for eligibility and final inclusion determined by consensus.

**Figure 2:**
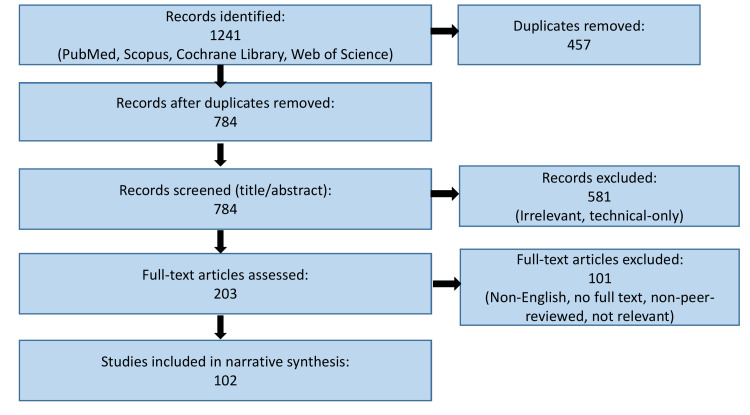
Flow diagram adapted from PRISMA principles to illustrate the literature identification and study selection process for this narrative review. PRISMA: Preferred Reporting Items for Systematic Reviews and Meta-Analyses

Although the manuscript is explicitly designed as a narrative review, elements of structured searching were employed to ensure comprehensive and unbiased coverage of the literature. The following flow diagram (Figure 2) is therefore adapted from Preferred Reporting Items for Systematic Reviews and Meta-Analyses (PRISMA) principles solely to improve clarity and transparency for readers, by illustrating how records were identified, screened, and included, without suggesting quantitative synthesis, risk-of-bias assessment, or systematic review rigor [11].

Common complications and their pathophysiology

Sarcopenia, a marker of impaired nutritional and functional reserve, has emerged as an important patient-related risk factor influencing outcomes after PD [12]. Preoperative sarcopenia has been consistently associated with higher rates of postoperative complications, including clinically relevant POPF, DGE, PPH, infectious complications, and prolonged hospital stay [13]. Proposed mechanisms include reduced tissue healing capacity, impaired immune response, increased frailty, and diminished physiologic resilience to surgical stress [12,13]. These patient-related factors interact with procedure-specific mechanisms discussed below.

Delayed Gastric Emptying

DGE is one of the most frequent complications after PD, with reported incidence ranging from 15% to 45%, depending on the definition and patient population [14]. The ISGPS standardized the definition in 2007, classifying DGE into three grades (A, B, and C) based on the need for nasogastric decompression, inability to tolerate oral intake, and the length of postoperative delay [8].

**Figure 3:**
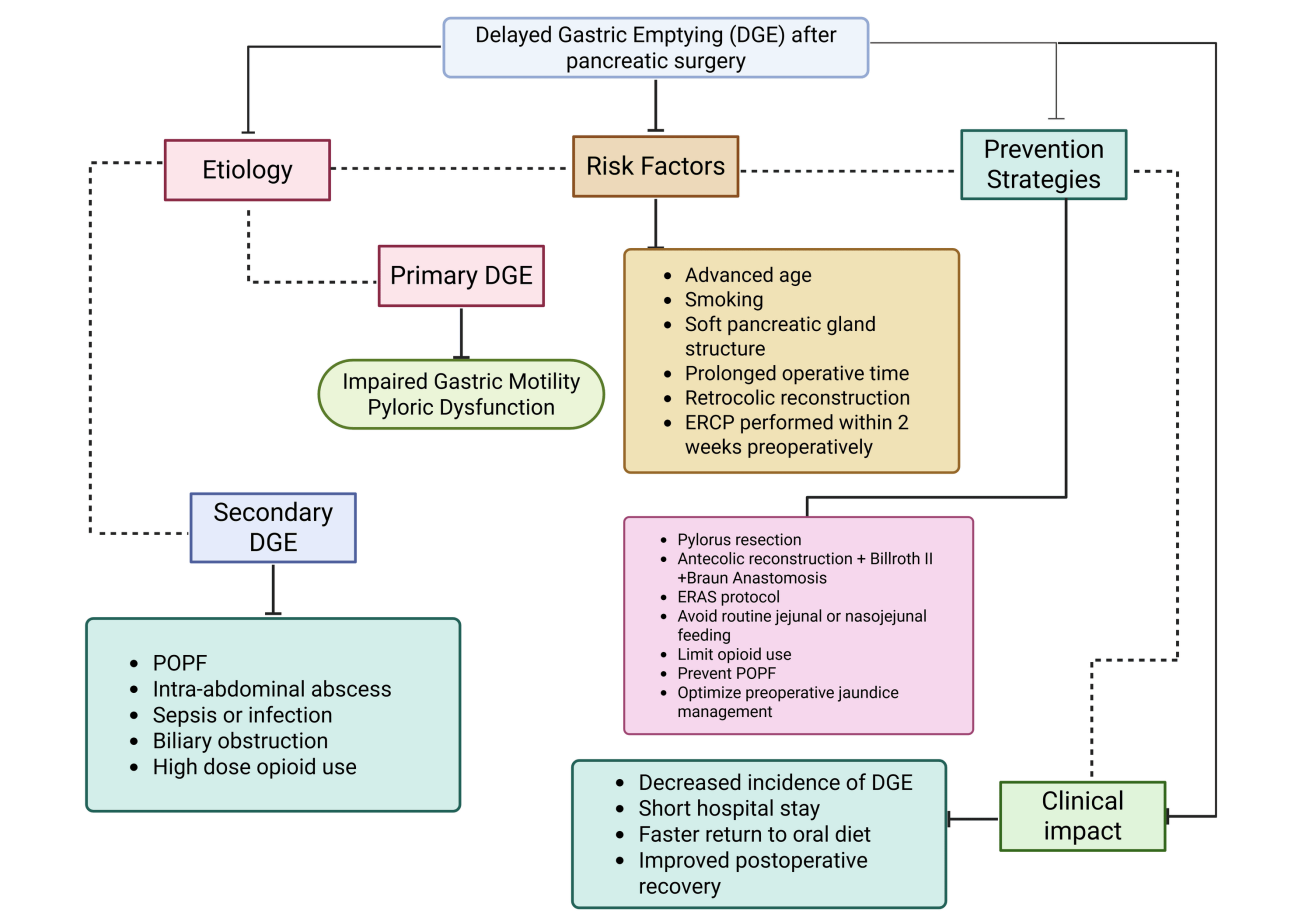
Visual representation of the multifactorial nature of delayed gastric emptying (DGE) after pancreatic surgery-etiologic pathways, patient/technical risk elements, preventive strategies, and clinical impact. ERAS: Early Recovery After Surgery; POPF: postoperative pancreatic fistula; ERCP: endoscopic retrograde cholangiopancreatography This is an original image created by the author Muzammil Qureshi

The pathogenesis of DGE is multifactorial and incompletely understood (Figure 3). Proposed mechanisms include disruption of the vagal nerve supply during surgery, alterations in gastrointestinal motility due to anastomotic reconstruction, and local inflammatory responses [15]. Additionally, postoperative complications such as intra-abdominal collections, pancreatic fistula, or infections may exacerbate DGE by inducing local inflammation or mechanical compression [16]. Hormonal and functional factors also play a role, with motilin suppression and impaired antral contractility observed in affected patients [17].

Risk factors include male sex, soft pancreatic texture, small pancreatic duct, and the presence of postoperative complications, particularly pancreatic fistula [18]. Reconstruction type (Billroth II vs. Roux-en-Y) and the use of pylorus-preserving vs. classical PD have also been investigated, but evidence remains inconclusive [19]. Clinically, DGE manifests as persistent nausea, vomiting, abdominal distension, and intolerance to oral feeding, often requiring prolonged nasogastric decompression and parenteral nutrition (PN) or enteral nutrition (EN) support [20].

Although DGE is rarely life-threatening, it prolongs hospital stay, increases healthcare costs, and negatively impacts recovery and quality of life [21]. A better understanding of its pathophysiology is crucial for developing preventive strategies, including enhanced surgical techniques, perioperative care optimization, and pharmacologic interventions targeting motility pathways.

Postoperative Pancreatic Fistula

POPF remains the most significant complication after PD, associated with increased morbidity, prolonged hospitalization, and risk of secondary complications such as intra-abdominal abscesses, hemorrhage, and sepsis [22]. The ISGPS updated its definition in 2016, describing POPF as a drain output of any measurable volume of fluid with amylase content greater than three times the upper limit of normal serum amylase, with clinically relevant grades B and C reflecting increased morbidity [5].

The pathophysiology of POPF is primarily related to failure of the pancreatic-enteric anastomosis. A soft pancreatic texture and a small (<3 mm) pancreatic duct are the most consistently reported risk factors [23]. Soft parenchyma is more prone to enzymatic leakage due to its fragile nature, while smaller ducts complicate the creation of a secure anastomosis [24]. High-output exocrine secretions rich in proteolytic enzymes further predispose to anastomotic disruption [25].

Additional risk factors include high intraoperative blood loss, long operative duration, obesity, and underlying pathologies such as ampullary or duodenal tumors, which are more often associated with soft glands compared to pancreatic adenocarcinoma [26]. Surgical technique (duct-to-mucosa vs. invagination anastomosis, use of stents, or sealants) and surgeon experience also influence fistula rates [27].

The sequelae of POPF are largely mediated through autodigestion and local inflammation. Leakage of pancreatic enzymes can erode adjacent vasculature, resulting in PPH, or promote intra-abdominal infection due to secondary contamination [28]. Understanding these mechanisms has fueled ongoing debates about optimal anastomotic techniques and prophylactic measures, such as somatostatin analogues or perioperative drains. Despite improvements in perioperative care, POPF remains a critical determinant of PD outcomes, highlighting the need for individualized risk stratification and tailored preventive strategies [29].

Postpancreatectomy Hemorrhage

PPH is an infrequent but potentially catastrophic complication after PD. The ISGPS classification-which considers timing (early ≤24 hours vs. late >24 hours), location (intraluminal vs. extraluminal), and severity-is widely used to guide diagnosis and management [30]. Early PPH typically reflects technical failures or inadequate intraoperative hemostasis, whereas late PPH most often results from vascular erosion driven by local inflammation, infected collections, or autodigestion from a pancreatic leak; late bleeds are frequently associated with pseudoaneurysm formation at vessels such as the gastroduodenal artery stump or hepatic artery branches [30].

Bile Leak

Bile leak after PD most commonly originates from the HJ (hepatic duct-jejunal anastomosis) or from injured small intrahepatic ducts. Reported incidence at high-volume centers is relatively low (commonly cited in the range of ~2%-8%), but when present, a bile leak markedly increases morbidity by promoting intra-abdominal collections, infection, and impaired healing of adjacent anastomoses [31,32].

Etiology is multifactorial and includes technical factors at the biliary anastomosis (tension, poor mucosal apposition, and marginal blood supply), small duct size, preoperative cholestasis or biliary inflammation, and secondary insult from adjacent leaks or infection (e.g., pancreatic fistula causing local inflammation). In fragile or inflamed ductal tissue, ischemia and suture line dehiscence become more likely [31,32]. Clinically, a bile leak presents with bilious drain output, elevated drain bilirubin relative to serum, abdominal pain, fever, or radiologic collections on computed tomography (CT) or ultrasound [31].

Intra-abdominal Abscess

Intra-abdominal abscess is a frequent and potentially severe complication following PD, often occurring as a secondary consequence of anastomotic leaks or POPF [33]. The incidence ranges between 10% and 20%, with variability depending on the definition and diagnostic criteria applied [34]. Abscesses typically form due to the accumulation of infected fluid collections at the surgical site, which may arise from undrained pancreatic or biliary secretions. The pathophysiology involves bacterial contamination of sterile intra-abdominal collections, often facilitated by impaired local immune responses and tissue ischemia [35].

Risk factors include the presence of POPF, bile leak, soft pancreatic texture, obesity, prolonged operative time, and inadequate drainage [36]. Clinically, patients present with fever, leukocytosis, abdominal pain, and sepsis in severe cases. Diagnostic imaging with contrast-enhanced CT is the gold standard, allowing identification of abscess cavities and guiding interventional drainage [37].

Surgical Site Infection

Surgical site infection (SSI) remains a common complication after PD, contributing significantly to morbidity, prolonged hospitalization, and increased healthcare costs [38]. The incidence ranges from 15% to 25%, despite advances in perioperative care and infection prevention protocols [39]. SSIs may be superficial, deep incisional, or organ/space-related, with the latter often overlapping with intra-abdominal abscesses [40].

The etiology is multifactorial, with wound contamination during surgery playing a central role. Risk factors include preoperative biliary drainage, obesity, malnutrition, diabetes, smoking, and prolonged operative duration [41]. The microbial spectrum frequently involves enteric Gram-negative bacteria (e.g., *Escherichia coli*), Gram-positive cocci, and, increasingly, multidrug-resistant organisms [42].

Pathophysiologically, impaired wound healing due to poor tissue perfusion, contamination by biliary or pancreatic secretions, and systemic immunosuppression increase the risk of SSI [43]. Diagnosis is clinical, based on erythema, discharge, or systemic inflammatory signs, supported by microbiological cultures. Effective SSI prevention and management remain vital in improving recovery trajectories after PD and reducing long-term morbidity.

Endocrine and Exocrine Pancreatic Insufficiency

**Figure 4:**
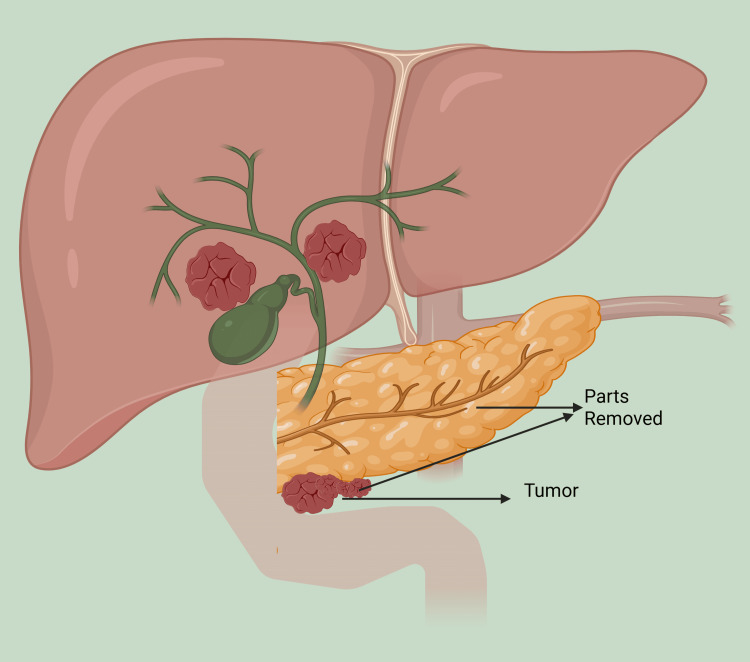
Complete pancreatectomy, highlighting the resected pancreatic tissue and its relation to adjacent structures. Image reproduced from Rizzo et al. [47] under the Creative Commons Attribution 4.0 International License (CC BY 4.0).

Endocrine and exocrine pancreatic insufficiency (EPI) are important long-term sequelae following PD, significantly impacting quality of life and long-term outcomes. EPI arises from loss of functional pancreatic tissue and altered gastrointestinal continuity, leading to insufficient secretion of digestive enzymes [44]. Incidence rates of EPI after PD are reported between 30% and 80%, depending on the extent of resection, underlying pathology, and assessment methods [45]. Symptoms include steatorrhea, weight loss, bloating, and fat-soluble vitamin deficiencies, which contribute to malnutrition and impaired postoperative recovery [46]. In select cases requiring completion or total pancreatectomy, such as extensive or multifocal intraductal papillary mucinous neoplasms (IPMNs), as illustrated in Figure 4, long-term risks such as diabetes and malabsorption become universal.

Endocrine insufficiency, most notably new-onset diabetes mellitus (NODM), occurs in 10%-40% of patients after PD [48]. The pathogenesis involves a combination of reduced β-cell mass, altered incretin hormone signaling, and postoperative insulin resistance [49]. Pre-existing chronic pancreatitis, higher body mass index, and resection for malignant disease have been identified as risk factors [50].

Chyle Leak

Chyle leak is a rare but significant complication following PD, characterized by the leakage of lymphatic fluid into the peritoneal cavity due to injury of retroperitoneal lymphatic channels during lymphadenectomy [51]. Its incidence ranges from 1% to 10%, with variability depending on diagnostic criteria and extent of nodal dissection [52].

The condition is typically defined as the presence of milky, triglyceride-rich fluid (>110 mg/dL triglyceride concentration) in abdominal drains after oral fat intake [53]. Pathophysiologically, disruption of the cisterna chyli or its tributaries leads to leakage of chyle, resulting in severe nutritional, metabolic, and immunological consequences [54]. Patients often present with persistent high-output drainage, electrolyte imbalance, weight loss, hypoalbuminemia, and increased susceptibility to infections [55].

Risk factors include extended lymphadenectomy involving clearance beyond standard peripancreatic nodal stations, including regions such as para-aortic and mesenteric root lymph nodes, resection for malignancy, and extensive retroperitoneal dissection [56]. Diagnosis relies on drain fluid analysis for triglyceride concentration and sometimes lipoprotein electrophoresis to confirm the presence of chylomicrons [57].

Management strategies for major complications

**Figure 5:**
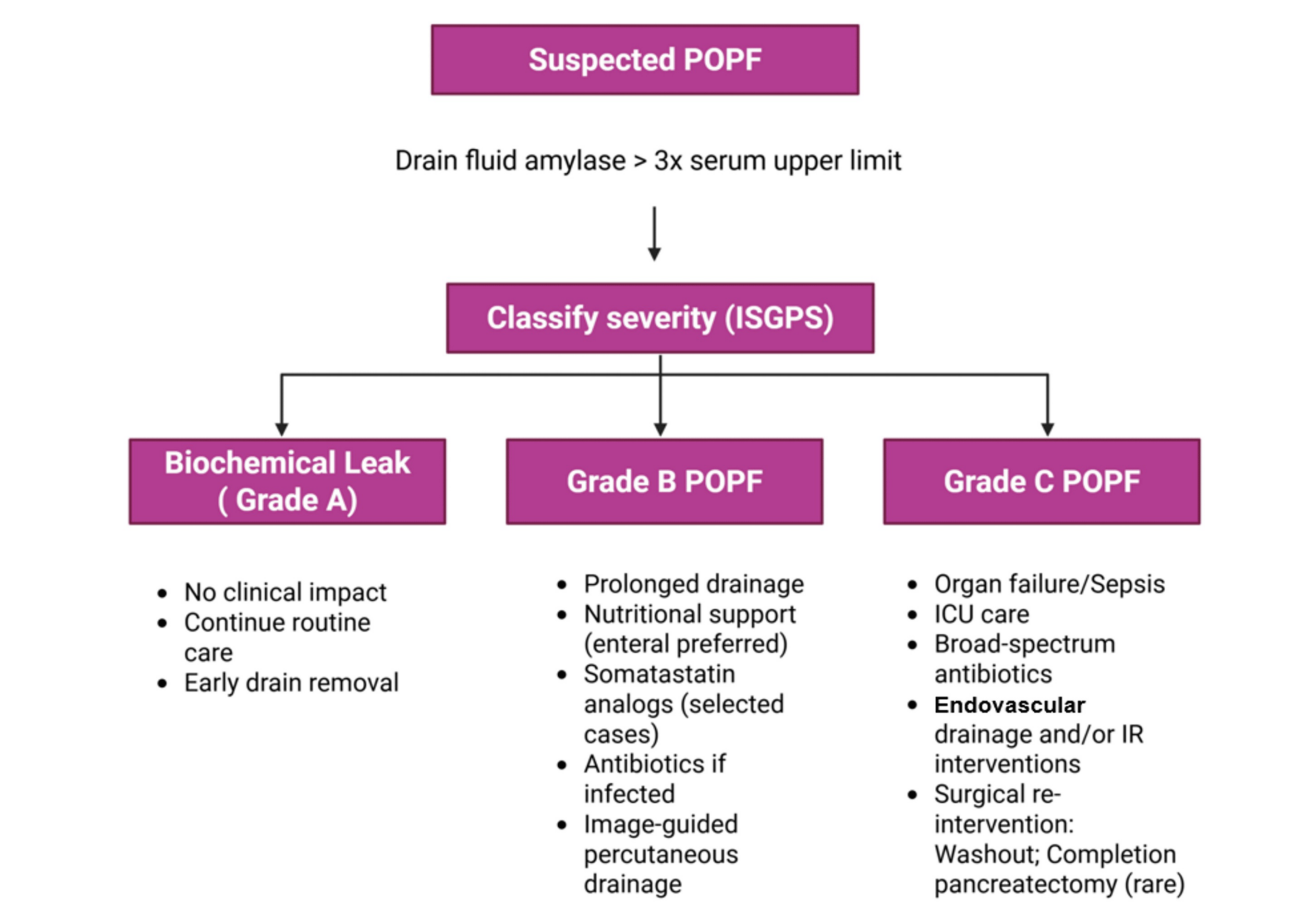
Stepwise management algorithm for postoperative pancreatic fistula (POPF). ISGPS: International Study Group of Pancreatic Surgery; ICU: intensive care unit; IR: interventional radiology Figure created in Biorender.com: https://app.biorender.com/illustrations/canvas-beta/69676e6cb824d1de3d10444c

**Figure 6:**
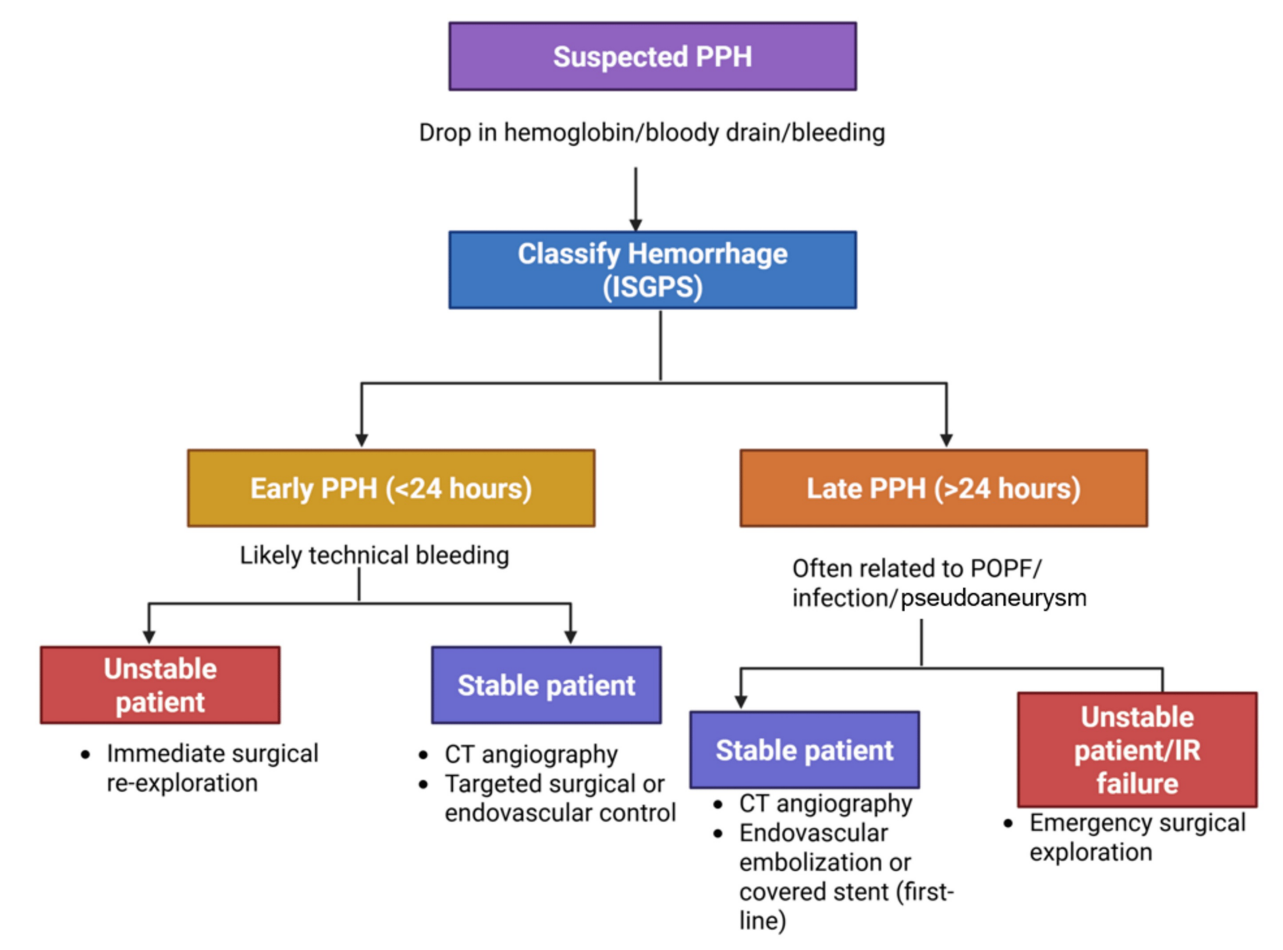
Stepwise management algorithm for postpancreatectomy hemorrhage (PPH). ISGPS: International Study Group of Pancreatic Surgery; CT: computed tomography; IR: interventional radiology; POPH: postoperative pancreatic fistula Figure created in Biorender.com: https://app.biorender.com/illustrations/canvas-beta/696774741b55aed31118ae05

Management of complications following PD requires a timely, structured, and multidisciplinary approach, integrating clinical assessment, radiologic evaluation, interventional techniques, and surgical decision-making. To provide an overview of contemporary, stepwise management pathways, Figures 5, 6 summarize evidence-based algorithms for the management of POPF and PPH, respectively.

Supportive Care

Supportive care remains primary in managing complications following PD, addressing nutrition, gastrointestinal motility, infection control, and fluid-electrolyte balance. Nutritional optimization is particularly critical, as malnutrition is both common and detrimental to recovery [58]. The controversy between EN and PN has long persisted. Several randomized controlled trials (RCTs) have demonstrated the safety and potential benefits of early EN compared to PN. In a multicenter RCT including 114 patients undergoing major pancreatic resection, Perinel et al. reported that EN was associated with lower infectious morbidity but higher rates of gastrointestinal intolerance [59]. Similarly, a large trial by Wheble et al. involving patients after gastrointestinal surgery demonstrated that PN was non-inferior to EN in terms of major outcomes, though EN remained associated with improved gut mucosal integrity and reduced septic complications [60]. Based on this evidence, current guidelines such as the European Society for Clinical Nutrition and Metabolism (ESPEN) and ERAS pathways recommend early EN, either oral or via enteral access such as nasojejunal or feeding jejunostomy routes, when feasible, reserving PN for patients with intolerance, DGE, or high-output fistulas [60,61].

In addition to nutrition, supportive care also encompasses prokinetic therapy for DGE. Clinical cohorts evaluating agents such as erythromycin and metoclopramide have demonstrated modest improvements in gastric emptying times, though without a significant impact on length of stay or mortality [62]. Infection control remains central, particularly in preventing progression of intra-abdominal collections or SSIs. Evidence from prospective cohorts indicates that early targeted antibiotic therapy guided by culture results improves outcomes compared with empirical broad-spectrum coverage [63]. Preoperative identification and optimization of sarcopenia through nutritional supplementation, resistance exercise, and prehabilitation programs may further reduce postoperative morbidity following PD [13]. Thus, supportive care in PD complications integrates nutritional strategies, symptom management, and rigorous infection control, forming the backbone upon which interventional and surgical measures build.

Interventional Radiology

IR has revolutionized the management of PD complications by offering minimally invasive, organ-preserving alternatives to reoperation. The most frequently employed technique is percutaneous drainage of intra-abdominal abscesses or collections, which has shown high clinical success. In a prospective cohort of 68 patients, Pham et al. reported that percutaneous drainage achieved resolution in 78% of post-PD abscesses, significantly reducing the need for surgical re-exploration [64]. Similarly, Zhou et al., in a cohort of 150 patients, found percutaneous drainage effective in controlling sepsis with lower morbidity and mortality compared to relaparotomy [65].

Endovascular embolization represents another key IR modality, primarily used in PPH. In a retrospective study of 108 patients with late PPH, embolization achieved hemostasis in 87%, though with a rebleeding rate of 15% [66]. A recent multicenter cohort study by Numoto et al. confirmed that endovascular management had significantly lower mortality than surgical intervention, particularly in hemodynamically stable patients [67]. Patient selection is crucial: while embolization is preferred for pseudoaneurysms and arterial bleeding, percutaneous drainage is prioritized for infected collections.

IR strategies are now considered first-line in many PD complications due to their minimally invasive nature, high success rates, and reduced perioperative morbidity. While surgical reintervention retains a role in unstable patients or failed IR, the cumulative data from cohorts consistently support IR as a safer and effective bridge, improving both short-term recovery and long-term survival.

Surgical Reintervention

Surgical reintervention after PD is reserved for a small but critically ill subset of patients in whom conservative measures and minimally invasive approaches have failed. Indications classically include refractory hemorrhage with hemodynamic instability, uncontrolled sepsis from anastomotic breakdown or infected collections not amenable to percutaneous drainage, and abdominal compartment syndrome [68]. Large single-center and multicenter cohorts report that the rate of unplanned reoperation after PD ranges from ~5% to 15% and that reoperation is associated with substantially higher morbidity and mortality than patients managed non-operatively, reflecting the severity of illness rather than the reoperation per se [69,70].

Key observational data help define timing and outcomes. Enderes et al. analyzed indications and outcomes of operative reintervention after PD in a tertiary center cohort and reported that early reoperations (within the first week) for technical failures (bleeding, anastomotic failure) had better salvage rates than delayed reoperations for sepsis and multiorgan failure; overall mortality after reoperation remained high (reported ~20%-30% in several series) [69]. A broader analysis of failure-to-rescue and reoperation showed that centers with lower failure-to-rescue rates achieved better survival after complications-highlighting the role of rapid recognition, escalation pathways, and intensive care unit (ICU) care in conjunction with surgery [69].

More focused analyses on reoperation for severe POPF/PPH suggest that the decision algorithm should prioritize endovascular and percutaneous approaches for hemorrhage and collections where feasible, reserving laparotomy for uncontrolled bleeding, bowel ischemia, or failure of IR measures. A recent systematic review of reoperation for POPF reported that when reoperation was required, it often involved source control maneuvers (drainage, lavage, debridement, and in some cases completion pancreatectomy or pancreatic transection) and that earlier intervention (before progression to irreversible multiorgan failure) correlated with improved survival [71].

The evidence (largely observational, with limited randomized data for reoperation timing) emphasizes patient selection and timing. Early, targeted reoperation for a discrete, surgically correctable problem (e.g., uncontrolled hemorrhage and anastomotic dehiscence amenable to repair) can be life-saving, while delayed reoperation in the context of systemic deterioration often has poor outcomes. Multidisciplinary pathways that combine rapid IR, critical care optimization, and timely surgical decision-making reduce failure-to-rescue and improve overall survival after PD complications [69,71].

Preventive Approaches

Prevention of major complications after PD focuses on surgeon-level technical modifications, standardized perioperative care pathways (ERAS), and pharmacologic adjuncts such as somatostatin analogues. High-quality randomized trials and large cohorts have tested these strategies with mixed but practice-informing results. Key surgical questions include the optimal pancreaticoenteric reconstruction (PG versus PJ) and the role of stenting or anastomotic reinforcement. The RECOPANC multicenter RCT (14 German centers) compared PG and PJ in high-volume centers and found no clear advantage of one technique over the other for clinically relevant POPF, suggesting that surgeon experience and standardized technique matter more than choice of anastomosis alone [72]. However, the technique of PJ itself significantly influences the risk of POPF. Commonly employed approaches include duct-to-mucosa anastomosis, invagination techniques, and binding PJ, with no single method demonstrating universal superiority across all patient populations. Randomized trials and large cohort studies suggest that outcomes are strongly influenced by pancreatic texture, duct diameter, and surgeon experience, rather than anastomotic technique alone [24,26,36]. As such, tailoring the PJ technique to gland characteristics, often combined with selective stenting or external drainage, represents a pragmatic strategy to reduce clinically relevant POPF.

Pharmacologic prevention has centered on somatostatin analogues. A single-center randomized trial published in NEJM (pasireotide) showed a reduction in clinically relevant pancreatic fistula and related complications in a high-risk cohort, prompting interest in broader use [29]. However, subsequent multicenter and larger cohort studies did not reproduce a consistent benefit, and a later randomized trial and meta-analyses tempered enthusiasm, concluding that routine prophylactic pasireotide or octreotide cannot be universally recommended and may be most useful in selected high-risk patients [73,74].

ERAS pathways for pancreatic surgery have been evaluated in multiple cohorts and meta-analyses. Systematic reviews show ERAS implementation reduces length of stay, opioid use, and some complication rates without increasing readmission, and ERAS elements (early mobilization, multimodal analgesia, restrictive fluid strategies, and early feeding) likely contribute to lower overall morbidity and faster functional recovery [75,76]. The randomized and cohort evidence supports adopting ERAS bundles rather than isolated elements.

Preventive strategies should be multimodal. Evidence supports (a) the use of standardized reconstruction techniques practiced in high-volume centers (operator expertise matters), (b) the selective use of somatostatin analogues-ideally targeted to patients at high risk for POPF rather than routine blanket use, and (c) full ERAS pathway implementation to reduce complications and hasten recovery. Adjunctive technical measures, including afferent loop decompression using nasojejunal or transanastomotic decompression tubes, have been shown to reduce intraluminal pressure at the pancreaticoenteric anastomosis and may lower the risk of POPF and DGE in selected patients [77]. Emerging adjuncts (anastomotic reinforcement, externalized stents in selected high-risk ducts) have promising cohort/RCT signals but require individualization based on duct size, gland texture, and institutional outcomes. Overall, the best prevention strategy is a systems approach: high surgical volume, standardized technique, ERAS implementation, and selective pharmacologic prophylaxis.

Pharmacological Strategies

Pharmacologic adjuncts in the peri- and postoperative period aim to reduce biochemical activity at anastomoses, manage acid-related complications, improve gastric motility, and prevent or treat infections. The literature contains randomized trials, meta-analyses, and cohort studies with sometimes conflicting results, so clinical use is often individualized.

Somatostatin analogues (octreotide, lanreotide, and pasireotide) have been extensively studied to reduce the incidence and clinical impact of POPF. Early randomized trials of octreotide suggested benefit in selected patients, but results were inconsistent [78]. The landmark single-center randomized, double-blind trial of pasireotide (300 patients) demonstrated a statistically significant reduction in clinically relevant POPF, leaks, and abscesses versus placebo, providing the strongest RCT signal in favor of a somatostatin analogue in a broad pancreatic resection population [79]. Subsequent meta-analyses and larger multisite evaluations tempered enthusiasm by highlighting heterogeneity in patient risk, variable effect sizes, and unclear cost-effectiveness when pasireotide is used indiscriminately; many groups now recommend selective rather than routine use, targeting high-risk patients (soft gland, small duct) rather than universal prophylaxis [76].

Proton pump inhibitors (PPIs) are commonly prescribed after PD to reduce marginal ulceration at gastrojejunal anastomoses and to control acid-related symptoms. Retrospective cohorts report reduced rates of marginal ulcers with routine PPI use but variable effects on short-term postoperative complications such as DGE or SSI; prospective randomized data are limited, and a contemporary clinical trial is ongoing to define perioperative PPI utility more clearly [80,81]. Given their safety profile, many centers continue short-term PPI prophylaxis, while weighing long-term risks (e.g., infections and micronutrient malabsorption) when prescribing prolonged courses.

Prokinetics (erythromycin, metoclopramide) are used to treat or prevent DGE. Randomized and cohort data show erythromycin (motilin agonist) accelerates gastric emptying and can reduce time to oral intake and nasogastric decompression in the early postoperative period, although benefits on hard outcomes (length of stay, mortality) are modest [82]. Metoclopramide and newer agents have less robust PD-specific trial data but remain reasonable choices when erythromycin intolerance or tachyphylaxis occurs.

Antibiotic stewardship is crucial: tailored prophylaxis (choice and duration) reduces surgical-site and organ-space infections without promoting unnecessary resistance. Recent prospective trials and cohort studies support shorter, targeted prophylaxis for patients without cholangitis, and one randomized/controlled antibiotic-duration study in PD after preoperative biliary drainage showed that limited-duration prophylaxis was non-inferior to prolonged courses in preventing SSIs when combined with drainage and surveillance [83]. A large comparative trial and contemporary institutional data have also suggested that using broader-spectrum agents (e.g., piperacillin-tazobactam) as single-dose perioperative prophylaxis may lower organ-space SSI in high-risk populations compared with older agents-though local microbiology should guide choices [84]. Implementing antimicrobial stewardship bundles (rectal screening, targeted prophylaxis, and perioperative audits) has improved SSI rates and reduced unnecessary broad-spectrum exposure in pancreatic surgery cohorts.

Hence, somatostatin analogues (notably pasireotide) show the strongest RCT evidence for reducing clinically relevant POPF but are best used selectively in high-risk patients. PPIs are commonly used to prevent marginal ulcers though high-quality perioperative trial data are limited. Erythromycin is effective for short-term management of DGE. Antibiotic strategy should be evidence-based, limited in duration when appropriate, and adapted to local biliary flora-stewardship interventions improve outcomes and reduce unnecessary exposure.

Minimally Invasive and Hybrid Approaches

Minimally invasive and hybrid techniques (laparoscopic, robotic, and combined IR/surgical approaches) have expanded beyond elective PD to selected situations in complication management and reintervention, with the aims of reducing physiologic insult and improving recovery in carefully chosen patients. Laparoscopic reintervention or minimally invasive drainage has been reported primarily in non-life-threatening indications (e.g., localized collections, symptomatic obstruction, and controlled fistula drainage). Contemporary single-center and multicenter cohorts indicate that when used in stable patients, laparoscopic drainage or targeted reoperation can achieve source control with less wound morbidity and shorter recovery than open relaparotomy [85,86]. Case series emphasize the importance of patient selection-small, well-localized collections or focal problems without diffuse peritonitis are better suited for a minimally invasive reapproach, whereas hemodynamic instability or diffuse sepsis still mandates open exploration [87].

Robotic surgery for index PD (robotic PD (RPD)) has matured, and registry/cohort data suggest comparable oncologic outcomes and, in some series, reduced blood loss and shorter length of stay compared with open PD in experienced centers [88,89]. This evolution supports performing selected reinterventions robotically where anatomy and expertise allow (for example, targeted adhesiolysis, localized abscess drainage, or revision of a pseudoaneurysm bed when IR is unsuitable). Recent international consensus documents on robotic pancreatic surgery recommend that robotic reinterventions be attempted only in centers with established RPD programs, with outcomes continuously audited [90].

Hybrid approaches integrate IR and minimally invasive surgery, for example, IR embolization for pseudoaneurysm plus laparoscopic drainage of infected collections, and several multicenter registries report improved organ preservation and lower reoperation rates when IR and minimally invasive surgery are used in a coordinated pathway [91]. Early data suggest shorter ICU stays and fewer wound complications, although long-term comparative randomized data are lacking, and selection bias in observational reports must be acknowledged.

Thus, minimally invasive and hybrid approaches offer real benefits (reduced wound morbidity, quicker recovery) for carefully selected PD patients with localized complications. The evidence base is predominantly cohort/registry data and single-center series; therefore, successful application depends heavily on institutional experience, multidisciplinary coordination (surgery + IR + critical care), and strict patient selection criteria. Open re-exploration remains the standard for unstable patients or diffuse intra-abdominal sepsis.

Table 1 provides an overview of the major complications after PD and evidence-based treatment strategies.

**Table 1:** Major complications after pancreaticoduodenectomy (PD) and evidence-based management strategies. NG: nasogastric; ERAS: Enhanced Recovery After Surgery; IR: interventional radiology; POPF: postoperative pancreatic fistula; ERCP: endoscopic retrograde cholangiopancreatography; MCT: medium-chain triglyceride; TPN: total parenteral nutrition

Complication	Management	Prevention
Delayed gastric emptying (DGE) [15-17]	• Supportive care: NG decompression, preference for enteral over parenteral nutrition • Prokinetics: erythromycin, metoclopramide • ERAS-based measures: early mobilization, early feeding	Minimize intra-abdominal complications; ERAS adherence improves outcomes
Postoperative pancreatic fistula (POPF) [22-26]	• Somatostatin analogues (pasireotide selectively for high-risk patients) • Drain management: early removal guided by amylase • Percutaneous drainage for collections • Surgical reintervention if uncontrolled	Centralization to high-volume centers; standardized surgical techniques reduce incidence
Postpancreatectomy hemorrhage (PPH) [30]	• Endovascular therapy: embolization (first-line for delayed bleeds) or covered stent-graft if hepatic flow preservation needed • Surgical re-exploration for unstable patients or IR failure	Meticulous intraoperative hemostasis; early detection and control of POPF/infection
Bile leak [31,32]	• Drainage: percutaneous or surgical • ERCP with biliary stenting in select cases • Surgical revision if major dehiscence	Careful biliary anastomosis; intraoperative leak testing where feasible
Intra-abdominal abscess [35-37]	• Percutaneous drainage (preferred) • Targeted antibiotic therapy • Surgical intervention if source control fails	Early recognition of POPF or bile leak reduces risk
Surgical site infection (SSI) [40-43]	• Perioperative prophylaxis: short, targeted antibiotics • Infection-control bundles: strict asepsis, wound care • Drainage/debridement for deep or persistent infections	Bundle compliance; optimize glycemic control
Endocrine insufficiency (new-onset diabetes mellitus, NODM) [44]	• Regular glycemic monitoring • Insulin therapy as indicated	Consider parenchyma-preserving procedures when oncologically safe
Exocrine pancreatic insufficiency (EPI) [45,46]	• Pancreatic enzyme replacement therapy (PERT) • Fat-soluble vitamin supplementation • Ongoing nutrition support with dietitian input	Long-term follow-up for adherence and dosing adequacy
Chyle leak [52-55]	• Dietary modification: low-fat/MCT diet, TPN if severe • Somatostatin analogues • Lymphangiography with embolization if refractory • Surgical ligation if leak point identified	Careful lymphatic dissection and intraoperative identification of leaks

Challenges and controversies

Despite significant advances in perioperative care, several controversies persist regarding outcome prediction and management following PD. A major challenge relates to definitional heterogeneity. Variability in how DGE and clinically relevant POPF are defined and reported continues to complicate interpretation. Although the ISGPS has established standardized definitions-DGE in 2007, graded A-C based on diet resumption and nasogastric decompression, and POPF in 2016, which reclassified “grade A” as a non-pathological “biochemical leak” and clarified grades B-C-many studies still employ older criteria alongside newer ones. This inconsistency hampers benchmarking, undermines meta-analyses, and may exaggerate intercohort differences. Systematic reviews confirm that outcome variability often reflects case-mix heterogeneity and divergent diagnostic thresholds rather than true differences in surgical performance [5,8,92,93].

The use of somatostatin analogues for prophylaxis remains another contentious issue. A Cochrane meta-analysis reported a modest reduction in fistula-related morbidity but inconsistent effects on endpoints such as reintervention or mortality, with trial heterogeneity precluding robust recommendations for universal prophylaxis [94]. More recent RCTs and meta-analyses, particularly those evaluating pasireotide, have produced conflicting results, highlighting the need for selective rather than routine prophylaxis, particularly in patients with high-risk anastomoses or soft pancreatic glands.

Drain management and enhanced recovery protocols also remain debated. The PANDRA trial demonstrated that omitting prophylactic drains did not increase POPF or reintervention and may reduce overall morbidity [95]. Subsequent RCTs and meta-analyses have supported risk-adjusted, early drain removal strategies-often guided by drain amylase levels-as safe and effective, leading to fewer complications, shorter hospitalization, and reduced costs [5,8,92,94]. At the same time, observational studies link excessive perioperative fluid administration to higher complication rates, providing support for more conservative fluid management under ERAS protocols, though questions of causality and generalizability persist [5].

Finally, the role of centralization and institutional volume continues to generate discussion. While data consistently associate high-volume centers with lower morbidity and mortality, attributable to surgeon expertise, multidisciplinary teams, and structured care pathways, such benefits are not universally attainable. In resource-constrained environments, centralization is often unfeasible, and extending minimally invasive PD beyond specialized centers poses additional challenges. Large multi-institutional registries underscore that standardized care pathways and specialist teams improve outcomes but also reveal the influence of selection bias and interinstitutional variability [96,97].

Future directions and research gaps

Despite notable progress in perioperative care, several avenues remain underexplored that could significantly advance the management of complications following PD. One promising area is the development of biomarkers for early detection of complications. Currently, reliance on clinical signs and biochemical markers such as drain amylase often delays intervention until complications are well established. Emerging research suggests that proteomic and genomic biomarkers, as well as inflammatory mediators, may provide earlier and more specific signals of impending fistula, hemorrhage, or infection [98-102]. Incorporating such tools into clinical practice could enable timely, individualized management and improve outcomes.

The application of artificial intelligence (AI) and predictive modeling represents another frontier. Machine learning algorithms using perioperative variables, imaging, and electronic health record data have demonstrated potential in predicting risks of POPF, DGE, and PPH with higher accuracy than traditional scoring systems [103,104]. However, these models require prospective validation across diverse patient populations and practice settings to ensure reproducibility and generalizability.

A persistent barrier to advancing research is the lack of uniform definitions and reporting standards. Although the ISGPS has provided consensus criteria for DGE, POPF, and other complications, inconsistent use continues to limit comparability between studies. Establishing mandatory adherence to standardized definitions in clinical research and registries is essential to allow valid benchmarking, meta-analyses, and evidence synthesis.

Finally, there is a clear need for prospective, adequately powered clinical trials to evaluate prophylactic strategies. Interventions such as somatostatin analogues, early drain removal, ERAS pathways, and anastomotic reinforcement techniques remain debated, with existing evidence often conflicting [5,76]. Future trials should incorporate stratification based on gland texture, duct size, and patient comorbidities to identify subgroups most likely to benefit.

## Conclusions

PD remains one of the most complex surgical procedures, with postoperative complications continuing to represent a major source of morbidity despite advances in surgical technique, perioperative care, and multidisciplinary management. Complications such as pancreatic fistula, DGE, hemorrhage, biliary and chyle leaks, intra-abdominal abscesses, and SSIs significantly influence short- and long-term outcomes, underscoring the need for timely recognition and tailored interventions. Progress in IR, minimally invasive reinterventions, and evidence-based perioperative strategies, including ERAS protocols and selective pharmacologic prophylaxis, has improved outcomes and reduced the need for high-risk surgical re-exploration. However, controversies remain regarding optimal prevention strategies, drain management, and prophylactic therapies. Future directions lie in the integration of predictive biomarkers, AI-driven risk models, and personalized approaches to patient care. Centralization to high-volume centers, adherence to standardized definitions, and further prospective research are critical to optimizing outcomes and reducing the burden of complications following PD.
